# The Development of Market-Driven Identities in Young People: A Socio-Ecological Evolutionary Approach

**DOI:** 10.3389/fpsyg.2021.623675

**Published:** 2021-06-22

**Authors:** Stephen Butler

**Affiliations:** Department of Psychology, University of Prince Edward Island, Charlottetown, PE, Canada

**Keywords:** advanced capitalism, adolescence, extrinsic values, market-driven identity, evolution

## Abstract

With the transition toward densely populated and urbanized market-based cultures over the past 200 years, young people’s development has been conditioned by the ascendancy of highly competitive skills-based labor markets that demand new forms of embodied capital (e.g., education) for young people to succeed. Life-history analysis reveals parental shifts toward greater investment in fewer children so parents can invest more in their children’s embodied capital for them to compete successfully. Concomitantly, the evolution of market-based capitalism has been associated with the rise of extrinsic values such as individualism, materialism and status-seeking, which have intensified over the last 40–50 years in consumer economies. The dominance of extrinsic values is consequential: when young people show disproportionate extrinsic relative to intrinsic values there is increased risk for mental health problems and poorer well-being. This paper hypothesizes that, concomitant with the macro-cultural promotion of extrinsic values, young people in advanced capitalism (AC) are obliged to develop an identity that is market-driven and embedded in self-narratives of success, status, and enhanced self-image. The prominence of extrinsic values in AC are synergistic with neuro-maturational and stage-salient developments of adolescence and embodied in prominent market-driven criterion such as physical attractiveness, displays of wealth and material success, and high (educational and extra-curricular) achievements. Cultural transmission of market-driven criterion is facilitated by evolutionary tendencies in young people to learn from older, successful and prestigious individuals (*prestige bias*) and to copy their peers. The paper concludes with an integrated socio-ecological evolutionary account of market-driven identities in young people, while highlighting methodological challenges that arise when attempting to bridge macro-cultural and individual development.

## Introduction

The challenge of identity in young people is perhaps more salient and formidable today than at any other time in history (Deci and Ryan, 2012). On the one hand, young people in advanced capitalist societies seem to have unparalleled opportunities to pursue and enact diverse identities, freed to a much greater degree from traditional structures and constraints (e.g., parental status, family codes and expectations, religious ideals) that historically channeled them into preordained roles. On the other hand, the diminished normative structures in advanced capitalism (AC) makes identity formation more complex and the passage to a functional adulthood more precarious (Sawyer et al., 2012; Coté, 2016). For example, young people face the tasks of securing access to higher education and an uncertain labor market, while developing an identity that will allow them to thrive in highly competitive and achievement-oriented capitalist cultures.

While young people in today’s capitalist cultures may be less influenced by traditional structures and previously held cultural beliefs and expectations as they develop their identities (Deci and Ryan, 2012; Coté, 2016), they nevertheless remain exposed to extensive social influences and pressures as they mature (Curran and Hill, 2019). The current paper proposes that, in AC societies, macro-cultural influences in the form of extrinsic values promoting physical attractiveness, wealth and material success, high achievements, social status and an enhanced self-image, exert significant effect on the development of young people’s identities. Concomitant with the cultural promotion of extrinsic values, young people are increasingly obliged to develop an identity that is market-driven and embedded in self-narratives of success, status, and enhanced self-image (Butler, 2018).

The construct of a market-driven identity offers a particular perspective on the socialization of young people and the disproportionate emphasis on macro-culturally promoted extrinsic values in AC. Researchers in areas such as materialism (Kasser, 2002), young people’s adjustment to high achieving schools (HAS; Luthar and Kumar, 2018), and self-determination theory (SDT; Deci and Ryan, 2012; Ryan et al., 2016) have all called attention to the extrinsic nature of AC and the corresponding challenges and mental health risks faced by young people. The challenges faced by young people of balancing extrinsic and intrinsic values in their emerging identities is consequential: when individuals show disproportionate extrinsic relative to intrinsic values in their overall value structure, there is increased risk for personal maladjustment and mental health problems (Kasser, 2002; Dittmar et al., 2014; Butler, 2018; Luthar and Kumar, 2018; Curran and Hill, 2019; Van Den Broeck et al., 2019). Therefore, it is imperative to promote healthy ecologies that will allow young people to cultivate a balance between intrinsic and extrinsic values while responding to AC market-driven pressures on their identify formation.

This paper further develops the hypothesis that young people in AC cultures are increasingly required to develop market-driven identities. First, definitions of advanced capitalism and identity are briefly presented. Second, evolutionary research is discussed to help establish key population-based parameters of market-based societies and their import for young people’s development. Complementing the use of evolutionary research, the next section briefly details similarities between the construct of market-driven identities in young people and historical precursors from the sociological literature. Once the evolutionary and historical foundations for the construct of market-driven identities in young people have been established, accumulating evidence is reviewed establishing status-seeking and extrinsic values such as individualism, materialism and interpersonal competitiveness as central to AC and the development of market-driven identities. These sections include consideration of how macro-culturally promoted extrinsic values are embodied by status and identity-enhancing market-driven criteria such as physical attractiveness, high achievements, and material success. Subsequently, integrating evolutionary and socio-ecological (Bronfenbrenner, 1979) perspectives, the paper suggests that young people signal these market-driven criteria in densely rewarding peer environments that are supported by neuro-maturational and stage-salient advancements of adolescence. That is, these developmental changes accentuate associations between young people’s developing identities and market-driven criteria, signaled in peer contexts where young people are biologically sensitive to peer evaluation and feedback, and status-attainment. The paper concludes with an integrative socio-ecological evolutionary model of market-driven identities in young people, while addressing the methodological challenges of developing empirically informed models that link macro-cultural and individual-level factors in young people’s development.

## Market-Driven Identities in Young People: Definitions

### Advanced Capitalism

Capitalism is an economic system based on private ownership of the means of production and their operation for profit (Zimbalist and Sherman, 2014). AC describes societies where the capitalist model has been developed deeply and extensively over a prolonged period of time. From the vantage point of young people’s psychosocial development, capitalism is an economic *and* social system that exerts broad and significant influences on how social relationships are organized and experienced (Deci and Ryan, 2012; Streeck, 2016). As such, the form of economic and social organization defined as AC is hypothesized to have psychological consequences for the developing identities of children and adolescents, which are of a deeply social nature (Brummelman and Thomaes, 2017). Research reviewed here is mainly from Western countries and includes studies that have been carried out in both liberal-market economies (LMEs; e.g., Australia, Canada, United States, United Kingdom) and more coordinated market economies (CMEs; e.g., Germany, Sweden, Denmark), a seminal distinction made by Hall and Soskice in their Varieties of Capitalism model (Hall and Soskice, 2001). The distinguishing feature of each type of economy is that they solve problems of ‘coordination’ (between the firm and its financiers, employees, suppliers, and customers) in different ways. In LMEs, coordination occurs primarily through market mechanisms, while in CMEs formal institutions play a much more central role in governing the economy and regulating firm relations with stakeholders. For example, in LMEs, wages are set by market forces, whilst in CMEs, they are determined through industry-level collective bargaining between employers’ associations and trade unions (Heery and Noon, 2008).

This paper attempts to integrate studies from evolutionary psychology and anthropology, neuroscience, and the socio-ecology of young people’s identity development and well-being. The theoretical framework adopted, articulated by Heine (2010), suggests that psychological processes arise from evolutionarily shaped biological potentials that become attuned to the particular cultural meaning systems that characterize AC, and therefore shape the development of young people. This framework does not reduce AC to a social or cultural construction, nor to a manifestation of evolutionary motives. Rather, it views AC as a dynamic socio-economic system that accentuates particular human potentials where culture and mind are mutually constituted (Heft, 2013).

### Adolescent Identity and SDT

The definition of adolescent identity is adapted from SDT, where identity is viewed as a developing self-representation of what and who the adolescent understands and describes themselves to be (Ryan et al., 2016). Identities are orienting for adolescents in that they provide a meaning-making lens and focus their attention on some but not other features of themselves and their immediate environment (Oyserman, 2007). Guided by extrinsic values, young people tend to adopt an outward orientation focused on impressing others by garnering external signs of self-worth (Williams et al., 2000). For example, young people’s self-displays of highly regarded consumer possessions or use of impression management to cultivate a selective, enhanced self-image online, exemplify the macro-cultural emphases on extrinsic values where money, good looks, high achievements, and fame portray and validate the self (Kasser et al., 2007).

The developing identities of young people require environmental affordances, supports, and feedback (Deci and Ryan, 2012). The psychosocial environments that support the development of market-driven identities in young people are often marked by status-seeking and identity-enhancing social interactions informed by emphases on extrinsically oriented values. For instance, recent research suggests that social media activities often foster psychosocial environments where many young people feel pressured to adopt extrinsic ideals and rely on others to validate their developing identities, values, and self-worth (Manago et al., 2008; Nesi and Prinstein, 2019). When young people base their developing identities on evaluations related to these extrinsic value targets, their self-image can become contingent on meeting them (Campbell, 1990; Kernis, 2003; Patrick et al., 2004).

Self-determination theory proposes that the controlling characteristics of family, socio-economic and national environments in AC predict a focus on extrinsic values while undermining the development of intrinsic ones (Deci and Ryan, 2012; Ryan et al., 2019). Identity pressures on young people in the form of extrinsic values from parents, peers and the broader macro-cultural environment (e.g., advertising, entertainment media and celebrity culture; social media), can become incongruent with young people’s own interests and sensibilities threatening the development of an integrated identity (Ryan and Deci, 2000; Kasser, 2002; Dittmar, 2007; Ryan et al., 2016). For example, regarding appearance ideals, Vartanian’s identity disruption model suggests that negative early life experiences in the form of compromised caregiving environments lead to body dissatisfaction and disordered eating, via lack of self-concept clarity which makes certain young people more susceptible to sociocultural factors such as social comparison and thin-ideal internalization when confronted with idealized images of female attractiveness (Vartanian and Hayward, 2018). Congruent with SDT, female adolescents who lack a clear sense of their own personal identity may be particularly vulnerable to internalizing market-driven criterion such as physical attractiveness as a means of self-definition (Campbell, 1990; Hogg, 2007).

Similarly, materialism research with young people suggests that overinvestment in materialistic values and status-oriented possessions tends to thwart the development of identity integration in young people. Young people who endorse high levels of materialistic values are more likely to conform to the expectations and prescriptions of significant others, potentially foreclosing identity exploration and the process whereby they engage in critical thinking about their own beliefs and values (Dittmar, 2007). Young people report higher levels of materialism when their parents (Kasser et al., 1995; Goldberg et al., 2003) and peers (Sheldon et al., 2000) are materialistic. They have also been shown to respond to peer pressure to become more materialistic (Banerjee and Dittmar, 2008; Rhee and Johnson, 2012), and compare themselves unfavorably to peers who have valued possessions relating to social acceptance and status (Roper and Shah, 2007). Studies of young people and materialism are consistent with young people pursuing self-enhancement rather than identity exploration, documenting relationships between high levels of consumption and materialism associated with feelings of insecurity, lack of self-concept clarity, and a desire for social status (Elliott and Leonard, 2004; Roper and Shah, 2007; Gil et al., 2012).

As few people adopt an all-extrinsic or all-intrinsic value orientation, one central issue for young people’s identity formation is the degree to which they will come to privilege extrinsic values in relation to intrinsic values in their overall value structure, rather than the absolute importance of either category (Vansteenkiste et al., 2008). Since status and identity-enhancing extrinsic values are so pervasive in AC cultures (e.g., Kasser et al., 2007; Schwartz, 2007; Greenfield, 2009; Twenge and Kasser, 2013), this paper argues that young people are required to develop an identity that situates their developing selves within this macro-cultural context. The construct of a market-driven identity is compatible with emerging concerns in the developmental and mental-health literatures that young people’s evolving sense of self in AC is pre-occupied with image-enhancement (Kasser, 2002; Côté, 2018; Luthar and Kumar, 2018), where the self is often portrayed as idealized (Dittmar, 2007) or perfectible (Verhaeghe, 2014; Curran and Hill, 2019). Congruent with the ascendancy of the idealized, perfectible, or perhaps *optimized* self, young people in AC have become preoccupied with upward social comparison and the need to define their social standing in relation to others (e.g., Richins, 1995; Myers and Crowther, 2009; Vogel et al., 2015; Fardouly et al., 2017; Curran and Hill, 2019).

Before proceeding further, it is important to acknowledge that identity is a rich and complex construct that can be difficult to delimit and assess empirically (Campbell et al., 1996). Across the diverse studies reviewed here, identity sometimes is defined by measuring associated or overlapping constructs, such as self-concept. More broadly speaking, identity theorizing is mostly based on verbal responses of participants and, as such, may not be a primary concern of evolutionary theorists. However, recent evolutionary research suggests that maturational processes such as identity formation are contextualized by competitive peer processes and broader cultural contexts (Worthman and Trang, 2018; Mesoudi, 2019), where young people are motivated to engage in cultural learning to help them negotiate the challenges of self-definition as they mature (Barkow et al., 2012; Mesoudi et al., 2016). In doing so, independent social behaviors and abstract cognition provide an ecological fit for the complex social worlds of AC (Greenfield, 2009), and these psychosocial capacities will promote young people’s adaptive plasticity to help them negotiate cultural influences that condition their identity formation (Sterelny et al., 2012). In AC, identity may be seen as an evolving self-narrative that is culturally shared and that implicates young people’s relation to market-driven criterion (e.g., how smart, attractive, or successful am I?). This culturally shared practice of establishing and elaborating identity is intimately tied to its communicative contexts, such as young people’s status and identity enhancing signaling afforded by status-enhancing consumer possessions or exchanges on social media.

## Market-Driven Identities in Young People: Building Competitive Young People

The delineation of advanced capitalism informed by recent evolutionary research helps elucidate the socio-ecological contexts that have evolved to promote young people’s development. The use of evolutionary research to characterize the transition from pre-industrial to market-based societies incorporates population-level developments and parameters that influence young people’s maturation. The evolutionary sciences are one of the fastest growing fields in the social and behavioral sciences (Mesoudi, 2019) and their impact when studying mental health and well-being is just beginning (e.g., Granqvist, 2020; Luyten et al., 2020; Schaller, 2020). Consequently, the application of evolutionary research to help link population patterns of change to individual-level psychological developments in young people such as identity-formation is tentative and preliminary.

One of the most striking developments with the transition to market-based industrial societies has been dramatic declines in fertility, initially in Western Europe and now throughout most of the world (Colleran et al., 2015, for review see Morita, 2018). From an evolutionary perspective, declines in fertility associated with the elaboration of market-based capitalism is a conundrum, given that evolutionary theory predicts organisms should maximize their reproductive success (Kaplan, 1996; Lawson and Mace, 2010; Colleran, 2016). While elucidating the complex causes of fertility decline in the demographic transition is beyond the scope of this paper, ongoing changes in reproduction have been linked to dramatic increases in the demands for investments in young people’s *embodied capital* (e.g., physical health, intelligence, knowledge and skills, social status and networks; Lancaster and Kaplan, 2010) and the related costs of raising children (Bongaarts, 2009; Shenk et al., 2016). In modern market-based societies, skills-based competitive labor markets increase the value of parental investment in children and motivate better-educated, higher income parents to invest more per child than their less-educated, lower-earning counterparts (Kaplan, 1996). Educational forms of embodied capital are believed to increase social status and therefore enhances an individual’s accessibility to resources (Morita, 2018).

Worthman and Trang (2018) document compulsory education as the radical innovation associated with the transition to market-based economies, occurring alongside shifts to competitive peer-dominated settings and adoption of an education-based wage labor economy. From an evolutionary life-history perspective, parents become implicated in a quantity-quality trade-off between number of offspring and the ability to invest in those offspring to maximize their chances for future success (Lawson and Mace, 2010; Giudice et al., 2015). Cultural shifts toward greater parental investment in fewer children in the transition to modern industrial societies allows parents to provide their children with opportunities to develop their embodied capital, and for children to profit from parental resources that will increase their chances to succeed (Kaplan, 1996; Worthman and Trang, 2018). Increased population density and urbanization in market-based capitalism, contemporaneous with decreased extrinsic mortality owing to improvements in public health (Lancaster and Kaplan, 2010), also support the shift toward greater investment in fewer children indirectly through greater number of competitors. The increased number of competitors elevates the benefits of reproducing later and less often, because organisms and their offspring need to invest more in embodied capital to compete successfully (Frankenhuis and Nettle, 2020). As a corollary, parents strive to maintain their own high socio-economic status so as to be able to provide high levels of investment in their children (Kaplan, 1996; Morita, 2018).

Young people’s development in AC is also occurring under conditions of greater social and occupational complexity (Greenfield, 2009; Sterelny et al., 2012). The elongation of adolescence (Sawyer et al., 2012), marked by prolonged and standardized schooling, lengthens the window devoted to embodied capital formation in an effort to increase young people’s capacity to gain employment and economic standing (Worthman and Trang, 2018). In navigating social and occupational challenges endemic to competitive, market-based AC, young people are acquiring less of their information and less of their values and goals from their parents and their parents’ close associates within the community, and more from their peers and figures of cultural prestige such as celebrities, through culturally promoted technologies such as television, advertising, and social media (Barkow et al., 2012; Jiménez and Mesoudi, 2019; Mesoudi, 2019; see prestige bias learning below). Consequently, while young people require greater informational resources to negotiate the complex social worlds of AC, the traditional social structures and networks that previously helped them integrate cultural information into their developing identities are less available and less useful (Barkow et al., 2012; Côté, 2018). In AC cultures young people’s identity development depends on mechanisms of individual adaptive plasticity and these mechanisms are likely to be profoundly influenced by cultural factors (Sterelny et al., 2012). Individual plasticity allows young people greater flexibility when developing values and pursuing goals in an effort to meet cultural and environmental demands.

The development of young people’s market-driven identities in AC is shaped by culturally promoted extrinsic values such as individualism, materialism, interpersonal competition and the pursuit of status (Kasser and Ryan, 1993; Kasser and Kanner, 2004; Kasser et al., 2007; Schwartz, 2007; Greenfield, 2009, 2013; Twenge and Kasser, 2013; García et al., 2015; Shahrier et al., 2016). While the goal of prolonged investment in young people’s embodied capital is to help them develop into productive and successful adults (Kaplan et al., 2003; Lancaster and Kaplan, 2010), the knowledge and skill sets that are needed to successfully transition toward adult roles are less certain. Young people, their parents and the broader culture advance distinct yet overlapping extrinsic criteria of success: high achievements (educational and extra-curricular), material success or resources, physical attractiveness, and popularity. As detailed below, these extrinsic value criteria are believed to be at once evolutionary-based and market-driven. Market-driven extrinsic value criteria are stage-salient for adolescence (e.g., Elstad, 2010; Hamel et al., 2012), supported by neuro-maturational developments that underlie young people’s heightened sensitivity to peer contexts and responsiveness to cultural influences (Crone and Dahl, 2012), and are embedded throughout young people’s socio-ecologies. Humans attempt to optimize their desired life outcomes (Baltes, 1997; Sterelny et al., 2012) and during adolescence young people’s heightened sensitivity to extrinsic values and goals overshadows the attention and cognitive resources that may be deployed to incorporate intrinsic goal-pursuit centered on relationships and emotional well-being (Carstensen et al., 1999; Carstensen et al., 2003; Crone and Dahl, 2012). Young people’s focus on extrinsic markers of success is intimately tied to their social contexts, and therefore the construct of market-driven identities in young people emphasizes their status and identity-enhancing functions in relation to peer and broader social networks.

Finally, it is legitimate to ask how radically different young people’s situation in AC is compared to other periods throughout history. In their application of embodied capital theory to human evolution and history, Lancaster and Kaplan (2010) conclude that the human predicament in the last 200 years is unique, linked to skills-based labor markets that demand new forms of embodied capital based on education and training for individuals to be able to access resources to compete and succeed. As these evolutionary developments pertain to young people, Worthman and Trang (2018) suggest that the perception of escalating demands for parental and social investments in embodied capital, associated with changes in parenting and reproduction that contribute to widespread fertility declines in market-based economies, have remodeled childhood, adolescence, and early adulthood.

Concurrent with the changes in reproduction and parental investment, the ascendancy of extrinsic values such as individualism and materialism in AC have been associated with the cultural shift in capitalist economies from industrial production to consumerism approximately 200 years ago, which have intensified over the last 40–50 years (Twenge et al., 2010, 2012a). The commanding influence of extrinsic values in a highly competitive socio-economic context are linked to reconfigurations of young people’s psychosocial and interpersonal processes, technology and the ambient socio-ecological conditions that inform their development and their developing identities. The concept of market-driven identity in young people is an attempt to address these changes and to understand part of this remodeling of young people’s lives.

## Market-Driven Identities in Young People: Historical Precursors

There are historical precursors to the notion that market-based capitalism gives prominence to extrinsic values that become central to young people’s developing identities. American historian and sociologist Mumford (1944) suggested that with market-based capitalism people began to find personal satisfaction in their extrinsic pursuits of wealth accumulation, i.e., the Goods Life, rather than in their intrinsic pursuits of living well, i.e., the Good Life. Mumford critiques the power of the market to objectify and quantify human striving, and in doing so, to devalue intrinsic human pursuits and ideals that are much more difficult to measure, such as moral ideals, satisfying human relationships and community. In 1950, American sociologist David Riesman (Reisman et al., 2001) conceptualized a shift in the character of individuals that accompanied the progression in capitalist societies from industrial production to consumerism. This change in individual character was away from “inner-directedness,” where people internalized goals instilled by their elders, toward “other-directedness,” where individuals became much more sensitized to the expectations and preferences of others, obtaining their sense of direction and guidance to a much greater degree from their peers and from figures portrayed in mass media. It is noteworthy that Reisman linked the prominent rise of the other-directed character specifically to the “internationalizing” tendencies of industrialism, urbanization, and capitalism, rather than to changes in American culture *per se*.

Congruent with Reisman’s attention to mass media as a significant influence in the development of other-directedness, Frank’s (1998) *Conquest of the Cool*, detailed the power of the advertising industry to promote and shape identities grounded in consumer goods that embodied cultural trends, values, and beliefs. More recently, Côté (2018) proposed that young people in AC are no longer developing identities grounded in traditional meanings and meaning-generating structures (e.g., stable families) that foster longer term values and goals, rather they face the challenges of forming their identities in present-focused environments that stress individualization and that foreground extrinsic values and pursuits. These “identity societies” promote self-interest and draw on young people’s skills related to impression management and self-enhancement, making it difficult for them to develop an integrated identity and to meet the challenges of adulthood. In the identity societies of AC, young people’s personal agency is a critical factor in their ability to manage recurrent market-driven pressures that pull them toward the novelty and immediate rewards of short-term extrinsically oriented pursuits and values, and away from a sense of purpose that resonates with their intrinsic pursuits and values, inner potentials, talents, and skills.

## Market-Driven Identities in Young People: Centrality of Status-Seeking in AC

The purpose of this section is to apply primarily evolutionary research to help understand the prominence of status-seeking in AC. As with identity, social status is a complex construct that is conceptualized and measured in diverse ways. The most common feature across the many definitions of social status is that it defines a person’s position or rank in relation to others (Ridgeway and Walker, 1995). While measurement of status across studies in support of the model of market-driven identities in young people varies considerably, almost all studies measure status positionally (e.g., Schwartz, 2007; Colleran et al., 2015; Shenk et al., 2016; Nesi and Prinstein, 2019).

From an evolutionary standpoint, social status can be defined as relative access to contested resources within a social group (Von Rueden et al., 2008). Social status in human societies is often based on prestige (Henrich and Gil-White, 2001; Cheng et al., 2010). Humans defer to the prestigious individual to gain access to, and thus socially learn from the prestigious individual, and to acquire other benefits such as private and public goods (Von Rueden et al., 2008; Anderson and Kilduff, 2009). Prestigious individuals receive ‘freely conferred’ deference that is linked to others’ perceptions of them. Social status therefore becomes dependent not only on the ability of the prestigious individual to confer benefits, but also on their advertisement of those abilities (Von Rueden et al., 2008). An emerging consensus in the literature is to use social status and prestige synonymously or as closely related terms (Cheng and Tracy, 2013; Blader and Chen, 2014; Anderson et al., 2015; Jiménez and Mesoudi, 2019), and this guideline is adhered to in the material that follows.

The pursuit of status and admiration and respect of our peers have been deemed human universals (Anderson et al., 2015) and some evolutionary psychologists view status-seeking as a fundamental evolutionary motive (e.g., Barkow, 1992; Schaller et al., 2017). Throughout our evolutionary history there have been tremendous benefits to social rank: greater respect, greater access to resources such as food and potential mates, and greater ability to control one’s own outcomes and satisfy one’s own goals. Each of these benefits ultimately spelled greater reproductive success (Barkow et al., 1975; Hill, 1984). The status sensitivity of humans appears to be supported by neurobiological systems: an emerging consensus among fMRI and neurochemical studies show that there are distinct neural circuits activated by attention to social rank (Izuma et al., 2008; Zink et al., 2008; Chiao et al., 2009; Ly et al., 2011), while earlier research demonstrated that altering serotonin levels in primates influenced status attainment (Madsen, 1994). Current developmental and neuroscientific research suggest that adolescence is a pivotal time for status-seeking. Studies have demonstrated that adolescents show heightened biological sensitivity to and reliance on peer feedback, heightened sensitivity to social rewards, and are motivated to attain status and secure their position in peer hierarchies (Harter et al., 1996; Somerville, 2013). In their review of the neuroscience of adolescence, Crone and Dahl (2012) suggest that the pubertal surge in testosterone levels amplifies the motivational salience of social status in males and females, which may lead to a general increase in the motivation to be admired. The behavioral repertoire and reward learning that result from the neuro-maturational significance of social status is culturally variable and sensitive to cultural context. In market-based AC, the hypothesis is that young people learn to associate status attainment with specific extrinsically oriented market-driven criteria, and that these criteria are embedded in their socio-ecologies and tend to be rewarded by parents, peers, and institutions such as schools.

While status-seeking is an evolutionary motive that is emerging during adolescence, it’s impact on culture and human development is neither fixed nor pre-determined. In this respect, research in evolutionary psychology and evolutionary anthropology supports the hypothesis that status-seeking motives are central to market-based AC (Buss et al., 2001; Miller, 2009; Ferguson et al., 2011a; Abed et al., 2012; Griskevicius and Kendrick, 2013; Barkow, 2014). Evolutionary research is complemented by studies in the critical domain of education, and more specifically research over the past few decades centered on the health and well-being of adolescents who attend high achieving schools (Luthar et al., 2013, 2020; Luthar and Kumar, 2018; Spencer et al., 2018; Ebbert et al., 2019).

Examining the cultural evolution of values associated with selecting a marriage partner across a 57-year period from 1939 to 1996, Buss et al. (2001) found that both sexes attached increased importance to the extrinsic values of physical attractiveness and wealth, with the latter especially seen in men. Furthermore, several studies suggest that decreases in fertility and family size associated with the demographic transition to modern market-based societies has been associated with increased investment in social status (Colleran et al., 2015; Shenk et al., 2016). Based on modeling data on fertility declines worldwide, Shenk et al. (2016) conclude that the geographical areas marked by the greatest declines in fertility are those areas defined by modern labor markets with intense competition for jobs and an overwhelming diversity of consumer goods available to signal social status and well-being. These authors conclude that, as competition becomes more focused on social climbing, couples invest more in material goods and achieving social status thereby influencing how many children they have.

Parents’ trade-off between their own status, particularly through education and status-related consumption, and their investment in children is also part of the dominant reproductive dynamic in AC (Shenk et al., 2016). For example, the dramatic drops in family fertility and greater investment in children outlined above have occurred alongside increases in positive assortative mating, where a competitive marriage market has been linked to increases in female education and married female workforce participation (Greenwood et al., 2017; Milanovic, 2019). The centrality of status-seeking in the parent domain recurs throughout the young person’s social ecology. When parents invest in their own or their children’s education to increase access to resources, they are simultaneously securing social rank and favorable positions in social networks, local communities, and society as a whole (Shenk et al., 2016).

Commensurate with socio-ecological developments in the parent domain, young people are sensitive to the display of prestige markers of social status and success in competitive peer contexts. For example, young people’s drive for status is occurring under economic transformations in AC where increasing numbers of educated adolescents reinforce competition for a qualification arms race and later employment (Worthman and Trang, 2018). Research examining educational achievement and the well-being of adolescents who attend high-achieving schools, illustrates how extrinsic values and status-seeking shape a particular ecology devoted to building competitive and successful young people (Luthar et al., 2013; Luthar and Kumar, 2018; Ebbert et al., 2019). Luthar et al. (2013, p. 1529) conclude that the risks associated with poorer adolescent well-being in these high-achieving ecologies operate within “the pervasive emphasis in contemporary American culture, on maximizing personal status, and how this can threaten the well-being of individuals and of communities.” More recently, these researchers emphasize the middle-class financial “squeeze” and consequent parental anxieties. Under these socio-economic conditions, parents become anxious that less than optimum performance by their children will increase the chances of their children being left behind, thus failing to attain the level of status that they themselves have achieved (Luthar et al., 2020). Young people in these environments feel significant pressures to succeed at the highest levels in all of their pursuits, not just academically but also in athletics and other extra-curricular activities (Spencer et al., 2018).

Regarding appearance ideals, the intrasexual competition hypothesis (Ferguson et al., 2011a, b; Abed et al., 2012) suggests that the ultimate cause of body dissatisfaction is intense intrasexual competition for mates realized in the competitive peer ecologies of adolescence and young adulthood. The unusual high number of youthful and youthful-looking women, and the provision of recurrent images of attractive competitors, are two aspects of market-based AC cultures that may intensify peer competition and promote signaling amongst adolescent and young adult females around appearance ideals.

## Market-Driven Identities in Young People: Ascendancy of Extrinsic Values in AC

The previous sections draw on evolutionary research to help established the unique parameters and competitive conditions that govern young people’s developing identities in AC, describing status-seeking as a fundamental evolutionary motive while suggesting that extrinsic values and status-attainment may be accentuated and embedded in young people’s socio-ecologies. This section documents accumulating empirical evidence that extrinsic values emphasizing individualism, interpersonal competition and status-attainment predominate in AC and are part of young people’s socio-ecologies. A range of studies are drawn upon to support the hypothesis that extrinsic values are defining features of market-based AC. These include evolutionary studies (Shahrier et al., 2016), cross-national research on culture and individual values (Schwartz, 2007; Goerke and Pannenberg, 2015; Alderson and Katz-Gerro, 2016; Van Den Broeck et al., 2019), numerous studies from Greenfield’s theory of social change and human development in the context of increasing marketization across historical periods, across nations and within countries (Greenfield, 2013; Xu and Takeshi, 2014; García et al., 2015), and recent cohort studies by Twenge et al. (2010, 2012a) documenting changes toward increased extrinsic values and decreasing intrinsic values in young people over the past 40–50 years. These studies are complemented by ethnographic and qualitative research with young people documenting the competitive nature of AC (Demerath, 2009) and relevance of extrinsic values in their lives (Spencer et al., 2018).

In his cross-cultural analysis of 76 countries, Schwartz (2007) concludes that LME particularly show a profile of extrinsically oriented values associated with achievement, hierarchy and power, and status in human relationships. Greenfield (2009) documents worldwide cultural trends toward market-based capitalist societies where people predominantly live in cities, receive formal education, and have lives immersed in high levels of technology. Individualistic values compared to collectivistic values are seen to be a better ecological fit for these capitalist cultures. Examining trends in the Google Ngram Viewer indexing individualistic and materialistic values from 1800 to 2000 in American English books, Greenfield (2013) chronicles that growth in these extrinsic values has accompanied the development of capitalist economies such as the United States and United Kingdom. Importantly, significant growth in individualistic and materialistic values have also accompanied China’s rapidly developing market economy (Xu and Takeshi, 2014; Zeng and Greenfield, 2015), and have been associated with national increases in wealth, level of formal education, and urbanization (Zeng and Greenfield, 2015).

Assessing social value orientation via an experimental manipulation with 1002 respondents from three areas of Bangladesh characterized by varying degrees of market-based capitalism, Shahrier et al. (2016) found that people were more likely to show greater competitiveness and less prosociality with movement from rural to urban, capitalistic cultures. Drawing on the concept of gene-environment co-evolution, the authors suggest that competitiveness is a cultural trait that is socialized in capitalistic cultures, and, consistent with Greenfield’s model, associated with urbanization and education. Similar findings were reported when studying co-operation and competitiveness in children from rural and urban settings in Mexico in 1970 and 2010 (García et al., 2015). As predicted, children in either environment were more competitive 40 years later, while children living in more urban environments characterized by greater wealth, education and technology use were more competitive in their behavior than children living in more rural settings.

These cross-national and national studies demonstrate that AC is associated with extrinsic values centered on individualism, materialism, competitiveness and status-attainment. Accumulating research also indicates that these extrinsic values are an integral part of young people’s socio-ecologies. For instance, among large representative samples of U.S. high school and college students there is evidence of increased individualism (Twenge et al., 2012b), and materialism and goals related to the pursuit of money, fame, and an enhanced self-image over the past 40–50 years (Twenge et al., 2010, 2012a). In a pre-adolescent sample, fame was identified as the top cultural value, actively cultivated through social media use, providing young people with an audience to respond to and shape their desire for recognition and status attainment (Uhls and Greenfield, 2012). In AC societies then, a self-regarding individualism predominates, which involves people wanting to become distinguished and acquire status, especially through direct competition with others (Chirkov et al., 2003).

These shifts toward a greater emphasis on extrinsic values in AC, in turn, influence young people’s development and well-being (Greenfield, 2009, 2016; Kasser et al., 2014). In a recent study examining associations between individual value pursuit and well-being among 25, 442 individuals aged 18–30 from 58 countries, young adults were typically happier and more satisfied with their lives when they lived in countries where intrinsic values prevailed and/or when extrinsic values were deemed less important (Van Den Broeck et al., 2019). It is also noteworthy that the macro-cultural shifts toward individualistic and materialistic values seen in China’s unprecedent growth as a market economy, have rapidly become associated with changes in the socio-ecology of children’s development. Longitudinal data demonstrates that shyness was associated with social and academic achievement in a cohort of Chinese elementary school children assessed in 1990, yet, by 2002 was associated with peer rejection, school problems, and depression. Children’s shyness, then, seems much less adapted to the socio-ecology of China’s burgeoning market economy (Chen et al., 2005).

Alongside documented increases in extrinsic values in AC, young people’s identity formation has become embedded in interpersonal relationships where competition and status seeking are prevalent. Demerath’s (2009) ethnographic study of a Midwestern suburban high school identified high levels of competition and social comparison among students as operative contextual factors at work in affluent communities in the United States. In their qualitative analysis of a sample of affluent girls from two independent girl schools Grade 6–12, interviews gathered from young females, parents, and teachers converged in documenting a strong, pervasive culture of competition among students (Spencer et al., 2018). Furthermore, meta-analytic results demonstrate that social comparison processes are linked to young people’s endorsement of extrinsic values such as appearance ideals (Myers and Crowther, 2009), while also predicting endorsement of materialistic values in young adults over and above variables including SES, emotional uncertainty and self-esteem (Kim et al., 2017). Cross-national research evaluating relationships between income and subjective well-being from both LME and CME indicate that a person’s relative position or social status is of substantial importance for his or her subjective well-being (Goerke and Pannenberg, 2015; Alderson and Katz-Gerro, 2016), identifying social comparison and status-seeking as mediating mechanisms of adverse well-being outcomes (Buttrick et al., 2017 for review).

Finally, a growing body of empirical studies show that social comparison permeates an individual’s responses to advertising (Richins, 1995; Gulas and McKeage, 2000) and involvement in social media (Fardouly et al., 2017). In both LME and CME, significant levels of social comparison are associated with negative psychological outcomes related to young people’s involvement with these technologies (Manago et al., 2008; Myers and Crowther, 2009; de Vries and Kühne, 2015; Vogel et al., 2015; Fardouly et al., 2017). The emphasis on status attainment and competitive individualism that characterizes AC appears to have become prominent in young people’ use of social media. In this regard, young people’s use of social media is often self-focused and involves idealized self-presentations refracted through the lens of impression management in the service of identity enhancement and status attainment (Manago, 2014; Bartsch and Subrahmanyam, 2015; Nesi et al., 2018).

## Market-Driven Identities: Translation of Extrinsic Values Into Market-Driven Criteria

**FIGURE 1:**
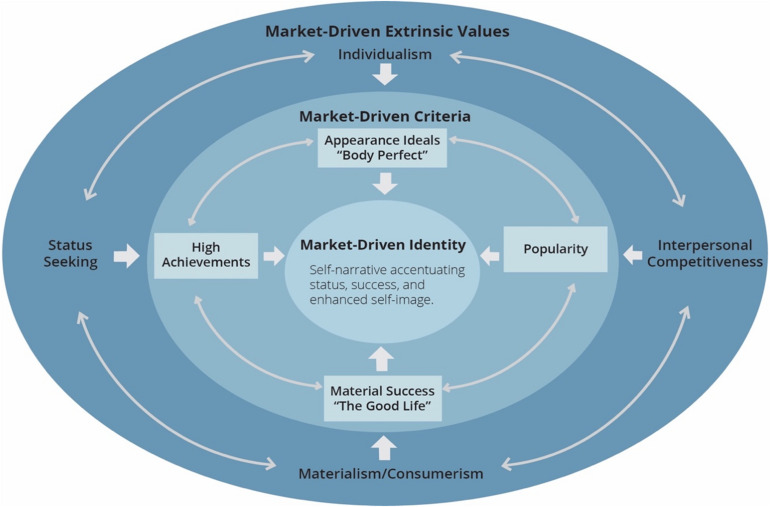
The macro-cultural context of market-driven identities in young people. Moving clockwise, the outer circle illustrates key market-driven extrinsic values, which are embodied by specific market-driven criteria, shown in the inner circle. These market-driven criteria, in turn, inform young people’s identity narratives, the circle at the center.

The previous sections established status-seeking and related macro-cultural extrinsic values such as competitive individualism and materialism as characteristic of market-based AC. This section details that these extrinsic values are embodied by market-driven criteria such as appearance and achievement ideals, and displays of consumer goods indicating wealth and success, that are part of young people’s identity formation (see Figure 1). Displays of market-driven criteria enhance young people’s self-image and serve to establish, reinforce, and extend their social status while providing them with a sense of belonging and self-worth. This section provides empirical support for the notion that prestige criteria are at once evolutionary based and market-driven.

Current research in both evolutionary psychology and young people’s mental health and well-being support the hypothesis that the extrinsic value structure of AC is associated with prominent market-driven criterion such as physical attractiveness or appearance ideals, displays of wealth and material success, and high achievements. For example, Bernard et al. (2005) provide an evolutionary-based typology of status motives in AC which include adaptations to improve one’s personal appearance through consumer displays or cosmetic surgery, to display one’s physique or wealth, and to promote one’s talents, abilities, or achievements. From an evolutionary framework, displays of these market-driven criteria enhance one’s status and desirability as a potential mate (Bernard et al., 2005). Status and identity-enhancing criteria rapidly become integrated into proximal symbolic and social systems as individuals develop in modern market-based societies (Barkow, 1989). There is substantial cross-national and country level-data in the areas of young people’s mental health and well-being that support the prominence of appearance ideals (Muth and Cash, 1997; Narring et al., 2004; Swami et al., 2010; Smink et al., 2012; British Association of Aesthetic Plastic Surgeons, 2015; PwC, 2015; American Society of Plastic Surgeons, 2016), displays of wealth and material possessions (Kunkel et al., 2004; Pew Research Center, 2007; Bailey, 2011; Jiang and Dunn, 2013; Parment, 2013; Bricker et al., 2014) and high achievements (West, 2010; Kearns, 2011; Thrupp, 2013; Wiborg, 2013; Banks and Smyth, 2015; Lingard and Lewis, 2016; Imsen et al., 2017) as critical extrinsic value markers in young people’s lives. The importance of educational achievement in the development of market-based industrial cultures has previously been noted (Kaplan et al., 2003; Worthman and Trang, 2018). This population-level research is complemented by selective studies of young people and their identities in areas such as appearance ideals (Patrick et al., 2004; Ashikali and Dittmar, 2012; Holsen et al., 2012; Easterbrook et al., 2014; Guðnadóttir and Garðarsdóttir, 2014; Daniels and Gillen, 2015; Kling et al., 2017; Vartanian and Hayward, 2018) materialism (Ashikali and Dittmar, 2012; Easterbrook et al., 2014; Guðnadóttir and Garðarsdóttir, 2014) and educational achievement (Wiklund et al., 2010; Deci and Ryan, 2012; Luthar et al., 2013; Luthar and Kumar, 2018; Spencer et al., 2018; Luthar et al., 2020), cited below or at salient points in the paper.

As noted above, there is accumulating cross-national and national data documenting that these market-driven criteria are characteristic of young people’s social-ecologies in AC. In terms of appearance ideals, levels of body dissatisfaction are high in both LME and CME; approximately 40% of young adult females and 20% of young adult males report body dissatisfaction in AC cultures such as the United States and Switzerland (Muth and Cash, 1997; Narring et al., 2004). In a study of body weight ideals and female body dissatisfaction in 26 countries across 10 world regions, body dissatisfaction and drive for thinness were most common in high-socioeconomic status AC countries (Swami et al., 2010). Recent cohort data from the United States and the United Kingdom show that incidence of body dysmorphia and eating disorders has risen by approximately 30% among late adolescent girls since the advent of social media (e.g., Smink et al., 2012; PwC, 2015). In the same countries, increasing numbers of young people are turning to plastic surgery and its promise of bodily perfection and image-enhancement (e.g., British Association of Aesthetic Plastic Surgeons, 2015; American Society of Plastic Surgeons, 2016). While rates of body dissatisfaction are high in both genders, the body is frequently characterized as a primary domain of female identity where female adolescents and adults are more likely to evaluate themselves and feel evaluated on the basis of their appearance (Daniels and Gillen, 2015; Kling et al., 2017).

Societal concerns over the increasing materialism and the commercialization of children’s lives is part of the fabric of AC, with reviews carried out by governments (e.g., Bailey, 2011, United Kingdom) and professional associations [e.g., Kunkel et al., 2004, American Psychological Association (APA)]. As noted earlier, materialistic values have been associated with the growth of market-economies in AC and evidenced in national and cross-national studies (Greenfield, 2009; Twenge et al., 2010; Twenge and Kasser, 2013). Data from the Pew Research Center (2007) suggests that more recent generations of young people borrow more heavily than did older generations at the same period of their life span, and, on average, spend a far greater proportion of their income on status possessions and image-enhancing goods than did their parents (Jiang and Dunn, 2013; Parment, 2013; Bricker et al., 2014). Materialism and conspicuous consumption are associated with identity enhancement through self and other signaling associated with displays of one’s possessions (Shrum et al., 2014).

There is convincing empirical evidence that appearance ideals and material success come together as status and identity-enhancing market-driven criteria in AC cultures. Dittmar (2007) argues that extrinsic values conferring social status to physical attractiveness (“Body Perfect”) and material success (“Good Life”) are two fundamental ideals of consumer culture. Individuals may become vulnerable to the adverse effects of these extrinsic ideals when they have become internalized. Consistent with this hypothesis, Ashikali and Dittmar (2012) found that priming materialism through experimental manipulation, namely exposure to advertisements of expensive, luxury goods, heightened the centrality of appearance to women’s views of themselves, and contributed to the activation of body-related self-discrepancies, especially for women high in self-reported materialism. Furthermore, studies with samples of children, adolescents and young adults of both genders from LME and CME suggest that extrinsic values associated with physical attractiveness and material success are mutually reinforcing components of identity in AC cultures (Easterbrook et al., 2014; Guðnadóttir and Garðarsdóttir, 2014).

Finally, the overwhelming importance of achievement ideals to young people’s education has received increasing attention. Most AC countries have adopted educational policies that enshrine high-stakes testing (HST) as a means to drive higher achievement in students, with the ultimate aim of fostering greater economic productivity (Rizvi and Lingard, 2010; Deci and Ryan, 2012; Lingard and Lewis, 2016). The development and consequences of high stakes testing for young people has been studied in LMEs such as the United States (Lingard and Lewis, 2016, United Kingdom; West, 2010, Australia; Klenowski and Wyatt-Smith, 2012; Thompson, 2013, New Zealand; Thrupp, 2013, Ireland; Banks and Smyth, 2015) and Canada (Kearns, 2011), and more recently in CMEs such as Sweden, Norway, and Denmark (Wiborg, 2013; Imsen et al., 2017).

The rapid growth and acceptance of government educational policies that privilege HST is partly rooted in extrinsic values characteristic of AC cultures (Ball, 2003; Rizvi and Lingard, 2010). In AC high-stakes educational environments, a competitive individualism tied to “objective” markers of success is embedded at all levels of the educational system (Deci and Ryan, 2012): schools are rewarded by governments for producing high achieving students with funding and high official rankings, while teachers, in turn, are rewarded by school administrators for their capacity to produce high achievers and meet government approved targets. Young people, their parents and teachers all make crucial links between high-stakes test performance and young people’s future prospects for lucrative employment and material success. It is understandable that parents and young people have focused their efforts and resources on maximizing high-stakes test performance (e.g., Alon and Tienda, 2007; Buchmann et al., 2010). The emphasis on high achievement and upward social comparison in young people’s educational performance accentuates well-established connections between high achievements, feeling of self-worth and the development of identity in young people (Elstad, 2010; Wiklund et al., 2010).

In summary, accumulating cross-national and generational research supports the predominance of extrinsic values in AC. This empirical evidence suggests a macro-culturally promoted set of values distinguished by individualism, materialism, and interpersonal styles rooted in competition. The hypothesis advanced here is that these values have become translated into market-driven criteria such as the pursuit of appearance ideals, high achievements and material success, and these criteria impinge on the formation of young people’s identities. The ultimate market-driven goal is to achieve success and attain status in the highly competitive and achievement-oriented cultures of AC (Luthar and Kumar, 2018; Spencer et al., 2018; Nesi and Prinstein, 2019).

## Market-Driven Identities in Young People: Cultural Transmission of Market-Driven Criteria

Humans possess a unique species-specific capacity to preserve, build-up and transmit socially learned information over successive generations (Dean et al., 2014). Adolescence is a sensitive period for specific forms of sociocultural learning and the acquisition of skillsets that are needed to transition successfully toward adult roles. In market-based cultures, the hypothesis is that young people’s identity formation has increasingly shifted toward extrinsic values and social contexts that promote social validation and status-seeking behavioral repertoires. Prestige learning *bias* in the evolution of human hierarchies may help us understand the socialization and intergenerational transmission of status-seeking motives and market-driven criteria in AC (Henrich and Gil-White, 2001). This hypothesis is underpinned by evolutionary theory and research on prestige bias learning (for review, Henrich and Gil-White, 2001; Barkow et al., 2012; Barkow, 2014; Mesoudi et al., 2016; Jiménez and Mesoudi, 2019; Mesoudi, 2019), as well as the impact of prestige figures such as celebrities on young people’s health and well-being via their prominent place in advertising (Miller, 2009; Knoll and Matthes, 2017), entertainment media (Niederkrotenthaler et al., 2012), and on social media (Jiménez-Castillo and Sánchez-Fernández, 2019).

Evolutionary theory posits that a major factor in human ecological success is our high-fidelity and selective social learning, which permits the accumulation of adaptive knowledge and skills over successive generations (Jiménez and Mesoudi, 2019). One strategy employed by humans to acquire adaptive social information is by preferentially copying competent individuals within a valuable domain, known as a success bias. However, given that competencies within a domain are often difficult or impossible to assess directly (e.g., what makes a successful partner), it has been hypothesized that individuals use indirect cues of success such as differential levels of attention paid to models by other social learners, as adaptive short-cuts to select models from whom to learn. This use of indirect markers of success, such as wealth, health or family size, is known as prestige bias (Henrich and Gil-White, 2001). It has also been suggested that prestige-based social learning is most salient during adolescence, when choice of identity and prestige criteria come into focus (Barkow, 2014).

Mesoudi (2019) describes three modes of cultural transmission characteristic of prestige *bias* learning: “vertical cultural transmission” (i.e., learning from one’s parents), “oblique cultural transmission” (i.e., learning from unrelated elders) and “horizontal cultural transmission” (i.e., learning from same-generation peers). Oblique and particularly horizontal transmission help account for rapid cultural change as they are not dependent on a limited number of role models (i.e., caregivers) and can occur within a generation. These two modes of cultural transmission have been associated with extrinsic values typical of Western AC societies, such as individualism (Mesoudi et al., 2016).

Oblique and horizontal transmission are particularly important during the developmental stages associated with identity formation, namely from late childhood through to early adulthood, following brief vertical transmission during early childhood (Mesoudi, 2019). The combination of vertical then oblique/horizontal transmission makes adaptive sense: initially copying one’s parents provides an initial guess at the appropriate knowledge for one’s environment, but this must then be updated by knowledge from others because one’s parents are too small a sample, who may not possess the full range of knowledge required to participate fully in society, and may possess out-of-date information (Mesoudi, 2019). This is particularly relevant to adolescence, where new domains involving sexuality, romantic love, moral dilemmas, and identity concerns require models to help young people develop new skills and navigate changing internal and external contexts.

The socialization of young people toward an extrinsic value orientation may partly be driven by their evolved tendencies to learn from successful and prestigious individuals (e.g., celebrities; *prestige bias*). Furthermore, oblique and horizontal cultural transmission may help account for the increasing prominence of extrinsic values from during adolescence, which are facilitated by young people’s susceptibility to peer influences and macro-cultural influences such as advertising and entertainment media, and their involvement in social media. Barkow et al. (2012) propose that in AC societies permeated by popular culture, young people are attending to and learning less from traditional role-models such as their own parents, family members and local celebrities, and more from high status celebrities and figures from entertainment and social media. These high-status ‘attractors’ orient young people’s identity-related processes toward market-driven criteria such as physical attractiveness, performing high achievements or displaying material wealth (Barkow et al., 2012).

Moreover, in the rapidly changing socio-ecological environment of AC, young people may confer prestige according to new values that give importance to skills that are currently relevant, and, in doing so, may select younger models from whom to learn (Jiménez and Mesoudi, 2019). In this respect, the emergence of one-sided emotional connections known as parasocial relationships, where a celebrity or internet star can feel quite proximate and personal, can be particularly strong during adolescence (Moreno and Uhls, 2019). Parasocial relationships benefit from prestige *bias* learning and often promote macro-culturally endorsed extrinsic values. For example, the use of celebrity endorsers is one of the most popular strategies used by traditional advertisers and internet platforms to promote brand allegiance and consumption. A recent meta-analysis on the effectiveness of celebrity endorsements in advertisements concluded that the most famous or high-status celebrities, who were also able to provide a strong credible product match-up, were the most efficacious (Knoll and Matthes, 2017). The emerging literature on digital influencers or the “new celebrities of today,” who are increasingly being used to endorse products and services online, also underscore their ability to embody elevated status or fame as one critical key to their power (Jiménez-Castillo and Sánchez-Fernández, 2019).

While prestige bias learning is consistent with the increasing relevance of non-familial models for adolescent identity formation, it is important to underline that prestige bias learning is not always adaptive. For example, a recent meta-analysis found that suicides of influential celebrities that are extensively reported in the media are more likely to result in increased suicides and a greater risk of a copycat effect (Niederkrotenthaler et al., 2012). Furthermore, the influence of high-status celebrities may sometimes lead to the acquisition of irrelevant or even maladaptive information via cross-domain prestige bias learning (Jiménez and Mesoudi, 2019). In this regard, young people’s attention to partly fictitious celebrities may direct them toward embracing appealing extrinsic value criteria that may interfere with developing more realistic and socially valuable strategies for accomplishment (Barkow et al., 2012). For example, studying marketing from an evolutionary perspective, Miller (2009) suggests that the images of wealth and beauty marshaled forth by advertisers to stimulate evolutionary-based motives to attract and keep mates, may undermine the importance of using more adaptive traits in decision-making about potential partners, such as intelligence and kindness that have been accentuated throughout evolution. Relatedly, in their study of American 12th graders values between 1976 and 2007 (*N* = 355,296), Twenge and Kasser (2013) found that while materialistic values increased, values associated with the importance of hard work steadily declined, suggesting a growing discrepancy between the desire for material rewards and the willingness to do the work usually required to earn them.

## Horizontal Cultural Transmission: The Peer Ecologies of Market-Driven Criteria

Adolescents themselves, like adults, are extremely concerned with their social position in relation to others, their self-image, and self-representations (Crone and Dahl, 2012; Gilbert, 2017). In this respect, there appears to be stage-salient compatibility between adolescence and the development of market-driven identities, tied to status-seeking motives exemplified by the prominence of extrinsic value criteria in AC cultures. It is within this particular stage-salient ecology that young people signal about themselves [e.g., as (sexually) attractive, high-achieving, wealthy or materially successful] to communicate about their status and success. This section draws on primarily empirical studies that demonstrate the status and identity enhancing functions of consumer displays (Elliott and Leonard, 2004; Roper and Shah, 2007; Dittmar and Bond, 2010; Gil et al., 2012; Furchheim et al., 2013; Shrum et al., 2013, 2014; Wang and Griskevicius, 2013; Durante and Griskevicius, 2016; Butler, 2018) and social media exchanges (Donath, 2002, 2007; Manago et al., 2008; Subrahmanyam et al., 2008; Zhao et al., 2008; Young, 2009; Dorethy et al., 2014; Fardouly and Vartanian, 2016; Liu et al., 2016; Nesi et al., 2018; Rai et al., 2018; Pangrazio, 2019).

In modern market-based contexts, signaling has social, symbolic and status-related aspects (Miller, 2013). Engaging in status and identity-enhancing signaling behavior, young people are attempting to strategically and successfully navigate complex psychosocial challenges in an increasingly competitive social and economic environment. The primary function of signaling is to enhance social status and its associated social and reproductive advantages (BliegeBird et al., 2005).

Young people’s displays of status-enhancing consumer goods or exchanges on social media occur in densely rewarding peer environments that promote status attainment. In adult studies, conspicuous consumption appears to involve “costly signaling” to enhance personal status and prestige through self-presentation (Furchheim et al., 2013; Wang and Griskevicius, 2013), and studies of children and adolescents’ consumer behavior suggest that their consumer displays may also reflect early manifestations of evolutionary drives to assert status and to promote affiliation through consumption (see Elliott and Leonard, 2004; Roper and Shah, 2007; Gil et al., 2012; Butler, 2018). Higher levels of materialism are indicated by greater acquisition and use of possessions to construct and maintain identity in young people and adults, due to signaling value of products and their identity-enhancing associations regarding wealth and status (Dittmar and Bond, 2010; Shrum et al., 2013, 2014; Durante and Griskevicius, 2016).

Most signaling that occurs in online peer environments can be conceptualized as “conventional” signaling, where the relationship between signal and its meaning exists by convention rather than a tightly structured relationship between the two (Donath, 2002). Conventional signaling may involve deception or the use of impression management strategies, as this can be quite beneficial to the signaler. Ultimately the receiver or the community at large arbitrates by imposing costs on dishonest signalers to maintain signal reliability, and to ensure that communication benefits both parties (Donath, 2002).

The digital spaces afforded by social media allow young people to selectively fashion self-narratives as they wish to be regarded by others, and to communicate this self-image easily and uniformly to a wide variety of others (Manago et al., 2008; Subrahmanyam et al., 2008; Liu et al., 2016). The prominence of status-seeking and identity enhancing signaling on social media is amplified by the speed, scale, range, and volume of social exchanges afforded by social media platforms (Nesi et al., 2018). Nesi and Prinstein (2019) advanced the notion of *digital status-seeking* whereby young people engage in a set of online behaviors (e.g., maximizing likes) to enhance their peer status and reputations. In their longitudinal study, adolescents with greater reputations for digital status seeking reported more frequent social media use and greater adherence to extrinsic value criteria such as desires for popularity and status attainment. In this densely rewarding communicative environment, market-driven criteria in domains such as popularity, physical attractiveness and achievement, provide a quantifiable and visually appealing system for measuring up how one stands in relation to other peers. On social media platforms like Facebook, ‘likes,’ photographs and ‘friends’ constitute a network of meaning that are key to a young person’s perceived social success, and central to understanding their market-driven identities (Pangrazio, 2019). In this respect, an emerging body of evidence, including longitudinal studies and experimental manipulations suggest that high levels of social media use is associated with increases in physical appearance concerns (see Fardouly and Vartanian, 2016 for review) and materialism (Ozimek et al., 2017; Rai et al., 2018), the former particularly in young females.

Studies suggest that young people engage in a range of impression management behaviors and strategies online that are status and identity-enhancing. For instance, to fulfill desires for social recognition, popularity, and fame, young people on social networking sites present themselves positively (Dorethy et al., 2014), disseminating images of them looking their best (Manago et al., 2008) and engaging in good times with friends (Zhao et al., 2008). Often, young people implicitly present themselves as socially desirable and use impression management techniques such as basking in reflected glory or self−promotion (Zhao et al., 2008), intentionally selecting photos for self−presentation (Sibak, 2009), particularly attractive ones (e.g., Young, 2009). Impression management strategies are also present when young people communicate information related to image-enhancement, such as status updates, counting friends, wall posts, and group membership (Zhao et al., 2008). In short, young people’s exchanges on social media often convey highly salient status and identity-enhancing images and associations related to market-driven criteria. These self-representations tend to be constructed to optimize their extrinsic appeal.

In summary, young people’s consumer displays and exchanges on social media afford for countless signaling opportunities in densely rewarding peer contexts. There is a growing consensus that social media affords for identity development that frequently involves impression management and the active curation of self-representations disseminated online. These self-representations are often embedded in market-driven extrinsic value criteria, where young people respond to cultural pressures to signal idealized or perfectible views of the self in a process of status and identity enhancement (Dittmar, 2007; Manago, 2014; Nesi and Prinstein, 2019). Neuroscientific findings underscore that these peer environments are primed for status and identity-enhancing signaling, given young people’s neuro-maturational sensitivities to peer evaluation and feedback, and the motivational salience to attain status and to be admired by their peers. In light of the current evidence, it may be that young people are not so much signaling ideal or perfectible selves, as much as *optimized* selves that benefit from persistent attention to status and image-enhancement in order to approximate and communicate the “best” version of oneself over time. The *optimized* self is therefore more of a dynamic, ongoing project, where young people curate and adjust their self-representations in relation to peer feedback, new life experiences and resources, or accommodation to one’s changing preferences and changing fashions.

## Summary and Conclusion

The purpose of this paper was to develop the hypothesis that young people in AC are increasingly obliged to develop market-driven identities, namely to foster self-representations that are predominantly linked to the expression of extrinsic values and corresponding self-narratives of success, status, and enhanced self-image (Butler, 2018). Substantial research shows that when individuals show disproportionate extrinsic relative to intrinsic values in their overall value structure, there is increased risk for mental health problems (e.g., Kasser, 2002; Dittmar et al., 2014; Luthar and Kumar, 2018). Consequently, the challenge faced by young people in balancing extrinsic and intrinsic values in their emerging identities is particularly important when growing up in highly competitive and achievement-oriented AC societies.

Evolutionary research suggests that, with the transition to densely populated and urbanized market-based cultures over the past 200 years, young people’s development has been inextricably linked to the ascendancy of skills-based labor markets that demand new forms of embodied capital based on education and training for humans to succeed (Kaplan et al., 2003; Lancaster and Kaplan, 2010). From a life-history perspective, parents are implicated in a quantity-quality trade-off between number of offspring and the ability to invest in those offspring to maximize their chances for future success (Lawson and Mace, 2010; Giudice et al., 2015). Cultural shifts toward greater parental investment in fewer children allows parents to provide their children with opportunities to develop their embodied capital, and for children to continue profiting from parental resources that will increase their chances to succeed (Kaplan, 1996; Worthman and Trang, 2018). Furthermore, increases in population density in market-based capitalism, contemporaneous with decreased extrinsic mortality owing to improvements in public health (Lancaster and Kaplan, 2010), support the wisdom of parents investing in fewer offspring and devoting their resources to enhancing their children’s embodied capital and ability to compete successfully (Frankenhuis and Nettle, 2020). Worthman and Trang (2018) argue persuasively that the perception of escalating demands for parental and social investments in embodied capital, associated with changes in parenting and reproduction in market-based economies, have remodeled childhood, adolescence, and early adulthood.

With the transition to large-scale and densely populated market-based capitalism, young people must negotiate complex social worlds that expand their informational requirements to make effective decisions. Young people’s individual adaptive plasticity is implicated in this decision-making under the profound influence of culture (Sterelny et al., 2012). The argument detailed throughout this paper is that extrinsic values embedded in the socio-ecologies of AC are defining culture influences in young people’s socialization and identify formation. At the population level, empirical evidence suggests that extrinsic values such as individualism, materialism, and status-seeking are characteristic of market-based economies (Greenfield, 2009, 2013) and have intensified in cohorts of young people over the last 40–50 years in consumer economies such as the United States (Twenge et al., 2010, 2012a).

Moreover, converging lines of inquiry highlight that macro-culturally promoted extrinsic values such as individualism, materialism and interpersonal competitiveness provide an ecological fit with market-based AC. At the individual level, emphases on status-seeking and extrinsic values in AC are synergistic with neuro-maturational and stage-salient developments of adolescence. Growing neuroscientific research suggests that important brain maturational changes are activated by puberty and continue during adolescence, when cortical and subcortical structures of the brain are re-organized and updated (Crone and Dahl, 2012; Blakemore, 2018). These neuro-maturational changes are associated with growth in young people’s social capacities, which include heightened sensitivity to peer contexts that involve social evaluation and feedback, and the search for and maintenance of social status, exemplified by the need to be admired (Crone and Dahl, 2012).

In market-based cultures, then, young people’s identity formation has increasingly shifted toward extrinsic values and socio-ecologies that promote status-seeking behavioral repertoires. Status attainment is associated with prominent market-driven criterion such as physical attractiveness or appearance ideals, displays of wealth and material success, and high educational and extra-curricular achievements. These status and identity-enhancing market-driven criterion have become integrated into symbolic and social systems as individuals develop in complex market-based societies (Barkow, 1989). Empirical literatures investigating market-driven criterion tend to employ a socio-ecological approach implicating the importance of parents, peers and broader macro-cultural factors when considering the potential consequences of each of these market-based criteria on young people’s mental health and well-being.

The integration of market-driven criteria into young people’s identity formation during adolescence is facilitated by the combination of oblique and horizontal cultural transmission. For instance, with the critical position that popular culture occupies in AC societies, and the diminished influence of normative structures such as family and communities, young people are learning less from traditional role-models and more from one-sided emotional connections related to high status celebrities and figures from entertainment and social media (Barkow et al., 2012; Moreno and Uhls, 2019). Moreover, peer exchanges on social media (e.g., Nesi and Prinstein, 2019) and displays of status-enhancing consumer goods (e.g., Griskevicius and Kendrick, 2013), provide densely rewarding peer environments for status attainment via displays of market-driven criteria. Alongside their status signaling functions, displays of macro-culturally promoted extrinsic value criterion are increasingly relied upon by many young people to obtain validation of their developing identities, values and self-worth (Manago, 2014).

Empirical research pertaining to market-driven criteria such as appearance ideals, material success and performing high achievements suggest that; (1) When young people base their developing identities on evaluations related to these market-driven criteria, their self-image can become contingent on meeting them (Campbell, 1990; Kernis, 2003; Patrick et al., 2004) and, (2) adolescents who lack a clear sense of their own personal identity may be particularly vulnerable to internalizing market-driven criterion as a means of self-definition (Campbell, 1990; Hogg, 2007; Vartanian and Hayward, 2018). One of the central issues for young people, then, is how they situate their developing identities in relation to these market-driven criteria.

On the one hand, in highly competitive and achievement-oriented AC environments, young people need to succeed and to situate their developing identities within market-driven AC criteria. As noted by Barkow (2014), part of the process of identity-formation for young people, including finding an employment or career trajectory, will involve trying out and discovering areas and domains that they can be successful in. At the same time, this exploration will occur within a socio-ecology where pressure to conform to market-driven criteria from parents and peers, particularly as these pursuits signal status-enhancement, may contribute to mental health difficulties and poorer well-being. For instance, when young people succeed at school or in athletics, the initial admiration and status attainment may help consolidate commitment and bolster the effort and drive needed to succeed. However, when young people’s pursuit of market-driven criteria becomes linked primarily to status-seeking and identity enhancement driven by pressures from their socio-ecology, they may be more likely to engage in high levels of social comparison with adverse consequences for their mental health and well-being.

Research suggests that an open and introspective identity stance facilitates young people’s self-determination and an information-oriented identity style would buffer against market-driven sociocultural pressures (Berzonsky, 2011). This identity style may also allow young people to reflect upon and consider the respective benefits and costs of pursuing these status and identity-enhancing ideals, and to consider their (emerging) place within their own lives. Recent studies have addressed the pressures of extrinsic values on young people’s well-being (Luthar and Kumar, 2018; Spencer et al., 2018), and include recommendations that encourage young people to cultivate intrinsic values and to develop a sense of purpose beyond their own achievements to encompass community ideals (Harvard University, 2020).

There are several methodological limitations to this review and the construct of market-driven identities in young people. First, it may be argued that a rigorous methodological approach that attempts to discover the impact of macro-cultural factors in AC on young people’s development requires comparisons between AC-cultures and non-AC cultures. Between-group comparisons would demonstrate conclusively that AC and non-AC cultures are truly different on independent variables such extrinsic values and market-driven criteria, which can then be related to identity formation outcomes. At the same time, the successful growth of market-based capitalist economies across the globe renders this type of comparison extremely difficult if not obsolete. This type of between-group comparison is arguably no longer feasible precisely because of capitalism’s uncanny ability to align macro-culturally promoted and individual-level objectives (Milanovic, 2019).

Nonetheless, the current paper has elaborated relationships between the macrosystem and young people’s identity formation by adhering to a rigorous methodological approach: privileging meta-analytic and longitudinal findings in the evidence-base hierarchy, incorporating studies that are well-suited to studying macro-cultural factors such as cross-national research and cohort designs, incorporating studies that use cultural evolutionary methods to examine variations in market-based economic development within countries in relation to extrinsic values such as social status and competitiveness, and, where available, considered distinctions within AC between LME and CME in Western nations.

To conclude, greater empirical work is needed to further develop the construct of market-driven identities in young people. For example, while the construct of market-driven identities finds general support across LME and CME, most studies reviewed here are from LME. Future research comparing outcomes between LME and CME would be beneficial, allowing for contrasts that have direct relevance to identity outcomes for young people. Moreover, future research that employs within-country variations in market-based socio-economic activity to study individual-level psychological variables would also be informative. Alternatively, it may be fruitful to test the construct of market-driven identities by comparing young people belonging to specific culture groups (e.g., Caucasian compared to indigenous groups or ethnic groups such as the Amish) that are believed to reflect within-society differences on key variables (e.g., levels of urbanization, individualism, materialism) to help test the fundamental tenets of the construct pertaining to status and identity enhancement. These types of studies would both increase our understanding of individual adaptation in relation to the macro-cultural context, and guard against interpreting population-level data in such a manner that supports the ecological fallacy.

To complement research examining market-driven identities in relation to key differences in social and cultural contexts, longitudinal studies would be necessary to help determine the socialization trajectory and long-term impact of market-driven identities in young people. It would be crucial for these samples to be studied as they transition into early adulthood, in order to study heterogeneity regarding the persistence of strongly held investments in extrinsic market driven criteria, or alternatively, young people’s shift toward greater perspective and balance in relation to intrinsic vs. extrinsic goals and values with accompanying outcomes. This program of longitudinal research could include cohort studies with already existing databases to determine whether in fact the emphasis on investment in extrinsic values and status-seeking follows a typical developmental progression such as peaking in mid-to-late adolescence and declining over the course of young adulthood, accompanied by greater emphases on intrinsic values and greater potential varieties in young people’s social ecologies (Crone and Dahl, 2012). Of course, heterogeneity in the prominence and course of market-driven identities would be expected.

Additionally, while the specific market-driven criteria identified in this paper are supported by cultural evolutionary and socio-ecological research, future development of the construct would benefit from closer examination of the developmental determinants of these market-driven criteria in young people’s identity formation. This would include mediators, such as social comparison and self-objectification, and moderators, such as gender, ethnicity, and social inequality. Finally, this review did not examine relationships between market driven criterion and social and racial inequalities, for example, the case of HST and educational attainment (Au, 2014).

It is hoped that the use of an evidence-informed framework to develop the notions of a market-driven identity in young people will encourage further consideration of the impact of the macro-cultural factors on young people’s identities and well-being. While studying the macro-cultural level can be perilous, there is an accumulating and substantial evidence-base that macro-cultural factors such as extrinsic values inform young people’s development and influence their mental health. Consequently, it may become increasingly perilous for psychology to ignore these developments.

## Data Availability Statement

The original contributions presented in the study are included in the article/Supplementary Material, further inquiries can be directed to the corresponding author.

## Author Contributions

The author confirms being the sole contributor of this work and has approved it for publication.

## Conflict of Interest

The author declares that the research was conducted in the absence of any commercial or financial relationships that could be construed as a potential conflict of interest.
