# Supplementary material for: The Development of Market-Driven Identities in Young People: A Socio-Ecological Evolutionary Approach
Source: Front Psychol. 2021 Jun 22;12:623675. doi: 10.3389/fpsyg.2021.623675 (PMC8258256; doi:10.3389/fpsyg.2021.623675)
Supplement: Supplementary file 1 [file Table_1.DOCX]

**Table 1: Hypothesis, component hypotheses and relevant literature**

| Hypothesis: **1a: Studies supporting the centrality of status-seeking motives in Advanced Capitalism** |
| --- |
| Sources: Evolutionary Psychology and Evolutionary Anthropology Studies of status-seeking and mating and reproduction  Buss, D. M., Shackelford, T. K., Kirkpatrick, L. A., & Larsen, R. J. (2001). A half century of mate preferences: The cultural evolution of values. *Journal of Marriage and Family*, *63*(2),491-503  Colleran, H., Jasienska, G., Nenko, I., Galbarczyk, A., & Mace, R. (2015). Fertility decline and the changing dynamics of wealth, status and inequality. *Proceedings of the Royal Society B: Biological Sciences*, *282*(1806), 20150287.  Shenk, M. K., Kaplan, H. S., & Hooper, P. L. (2016). Status competition, inequality, and fertility: implications for the demographic transition. *Philosophical Transactions of the Royal Society B: Biological Sciences*, *371*(1692) |
| **Hypothesis 1b: Studies supporting the importance of extrinsic values in Advanced Capitalism** |
| Sources: Market-based capitalism and extrinsic value-orientation  García, C., Rivera, N., & Greenfield, P. M. (2015). The decline of cooperation, the rise of competition: developmental effects of long‐term social change in Mexico. *International Journal of Psychology*, *50*(1), 6-11.  Greenfield, P. M. (2013). The changing psychology of culture from 1800 through 2000. *Psychological science*, *24*(9), 1722-1731.  Schwartz, S. H. (2007). Cultural and individual value correlates of capitalism: A comparative  analysis. *Psychological Inquiry*, *18*(1), 52-57.  Shahrier, S., Kotani, K., & Kakinaka, M. (2016). Social value orientation and capitalism in societies. *PLoS One*, *11*(10).  Xu, Yi, and Takeshi Hamamura. "Folk beliefs of cultural changes in China." *Frontiers in Psychology* 5 (2014): 1066.  Sources: Market-based capitalism and *intensification of extrinsic values* from roughly 1960/70-2010 (cohort studies)  Twenge, J. M., Campbell, W. K., & Freeman, E. C. (2012). Generational differences in young adults' life goals, concern for others, and civic orientation, 1966–2009. *Journal of personality and social psychology*, *102*(5), 1045.  Twenge, J. M., Campbell, S. M., Hoffman, B. J., & Lance, C. E. (2010). Generational differences in work  values: Leisure and extrinsic values increasing, social and intrinsic values decreasing. *Journal of management*,  *36*(5), 1117-1142.  Twenge, J. M., Campbell, W. K., & Gentile, B. (2012). Increases in individualistic words and phrases in American books, 1960–2008. *PloS one*, *7*(7).  Twenge, J. M., & Kasser, T. (2013). Generational changes in materialism and work centrality,1976-2007: Associations with temporal changes in societal insecurity and materialistic role modeling. *Personality and Social Psychology Bulletin*, *39*(7), 883-897 |
|  |

| **Hypothesis 2a: The importance of extrinsically market-driven criteria for young people’s identities** |
| --- |
| Sources: Physical appearance ideals, national and cross-national data from AC countries  Narring, F., Tschumper, A., Inderwildi Bonivento, L., Jeannin, A., Addor, V., Bütikofer, A.  ... & Michaud, P. A. (2004). SMASH 2002: Swiss multicenter adolescent survey on health 2002. *Lausanne: Institut universitaire de médecine sociale et préventive*  PwC. (2015). *The costs of eating disorders: Social, health and economic impacts*. Retrieved. from https://www.b-eat.co.uk/assets/000/000/302/The_costs_of_eating_disorders_Final_original.pdf  Swami, V., Frederick, D. A., Aavik, T., Alcalay, L., Allik, J., Anderson, D., ... & Danel, D. (2010). The attractive female body weight and female body dissatisfaction in 26 countries across 10 world regions: Results of the International Body Project I. *Personality and social psychology bulletin*, *36*(3), 309-325.  Smink, F. R., van Hoeken, D., & Hoek, H. W. (2012). Epidemiology of eating disorders: Incidence, prevalence and mortality rates. *Current Psychiatry Reports, 14,* 406 – 414. http://dx.doi.org/10.1007/s11920- 012-0282-y  Sources: Importance of Higher Educational Achievement in AC  *As Embodied Capital from an evolutionary perspective*:  Kaplan, H., Lancaster, J., & Robson, A. (2003). Embodied capital and the evolutionary economics of the human life span. *Population and Development Review*, *29*, 152-182.  Worthman, Carol M., and Kathy Trang. "Dynamics of body time, social time and life history at adolescence." *Nature* 554, no. 7693 (2018): 451-457.  *Pressures of high educational achievement in young people’s socio-ecologies across Western nations:*  Banks, J., & Smyth, E. (2015). ‘Your whole life depends on it’: academic stress and high-stakes testing in Ireland. *Journal of Youth Studies*, *18*(5), 598-616.  Imsen, G., Blossing, U., & Moos, L. (2017). Reshaping the Nordic education model in an era of efficiency. Changes in the comprehensive school project in Denmark, Norway, and Sweden since the millennium. *Scandinavian Journal of Educational Research*, *61*(5), 568-583.  Kearns, L. L. (2011). High-Stakes Standardized Testing & Marginalized Youth: An Examination of the Impact on Those Who Fail. *Canadian Journal of Education*, *34*(2), 112-130.  Klenowski, V., & Wyatt-Smith, C. (2012). The impact of high stakes testing: The Australian story. *Assessment in education: Principles, policy & practice*, *19*(1), 65-79.  Lingard, B., & Lewis, S. (2016). Globalisation of the Anglo-American approach to top-down, test-based educational accountability. *Handbook of human and social conditions in assessment*, 387-403.  Thrupp, M. (2013). National Standards for student achievement: Is New Zealand's idiosyncratic approach any better?. *Australian Journal of Language and Literacy, The*, *36*(2), 99.  West, A. (2010). High stakes testing, accountability, incentives and consequences in English schools. *Policy & politics*, *38*(1), 23-39.  Wiborg, S. (2013). Neo-liberalism and universal state education: The cases of Denmark, Norway and Sweden 1980–2011. *Comparative education*, *49*(4), 407-423.  Sources: The importance of material success in AC  Consumer goods as status-enhancing:  Bricker, J., Ramcharan, R., & Krimmel, J. (2014). *Signaling status: The impact of relative income on household consumption and financial decisions* (FEDS Working Paper 2014–76). Washington, DC: Federal Reserve Board.  Jiang, S. S., & Dunn, L. F. (2013). New evidence on credit card borrowing and repayment patterns. *Economic Inquiry, 51,* 394 – 407.  Parment, A. (2013). Generation Y vs. baby boomers: Shopping behavior, buyer involvement and implications for retailing. *Journal of Retailing and Consumer Services, 20,* 189–199. (cohort data)  Pew Research Center. (2007). *How young people view their lives, futures and politics: A portrait of “Generation Next”*. Retrieved from http:// people-press.org/report/300/a-portrait-of generation-next  Sources: Market-driven criteria and young people’s identity:  Ashikali, E. M., & Dittmar, H. (2012). The effect of priming materialism on women's responses to thin‐ideal  media. *British Journal of Social Psychology*, *51*(4), 514-533.  Barkow, J. H. (2014). Prestige and the ongoing process of culture revision. In *The psychology of social status* (pp. 29-45). Springer, New York, NY.  Dittmar, H., & Bond, R. (2010). I want it and I want it now: Using a temporal discounting paradigm to examine predictors of consumer impulsivity. *British Journal of Psychology*, *101*(4), 751-776  Easterbrook, M. J., Wright, M. L., Dittmar, H., & Banerjee, R. (2014). Consumer culture ideals, extrinsic  motivations, and well‐being in children. *European Journal of Social Psychology*, *44*(4), 349-359.  Elstad, J. I. (2010). Indirect health-related selection or social causation? Interpreting the educational differences in adolescent health behaviours. *Social Theory & Health*, *8*(2),134-150.  Guðnadóttir, U., & Garðarsdóttir, R. B. (2014). The influence of materialism and ideal body internalization  on body‐dissatisfaction and body‐shaping behaviors of young men and women: Support for the  Consumer Culture Impact Model. *Scandinavian Journal of Psychology*, *55*(2), 151-159.  Hogg, M. A. (2007). “Uncertainty—identity theory,” in *Advances in Experimental Social Psychology*, Vol. 39. ed M. P. Zanna (San Diego, CA: Elsevier Academic Press), 69–126.  Holsen, I., Jones, D. C., & Birkeland, M. S. (2012). Body image satisfaction among Norwegian adolescents and young adults: A longitudinal study of the influence of interpersonal relationships and BMI. *Body image*, *9*(2), 201-208.  Luthar, S. S., & Kumar, N. L. (2018). Youth in high-achieving schools: Challenges to mental health and directions for evidence-based interventions. In *Handbook of School-Based Mental Health Promotion* (pp. 441-458). Springer, Cham.  Patrick, H., Neighbors, C., & Knee, C. R. (2004). Appearance-related social comparisons: The role of contingent self-esteem and self-perceptions of attractiveness. *Personality and social psychology bulletin*, *30*(4), 501-514.  Spencer, R., Walsh, J., Liang, B., Mousseau, A. M. D., & Lund, T. J. (2018). Having it all? A qualitative examination of affluent adolescent girls’ perceptions of stress and their quests for success. *Journal of Adolescent Research*, *33*(1), 3-33.  Vartanian, L. R., Hayward, L. E., Smyth, J. M., Paxton, S. J., & Touyz, S. W. (2018). Risk and resiliency factors related to body dissatisfaction and disordered eating: The identity disruption model. *International Journal of Eating Disorders*, *51*(4), 322-330.  Wiklund, M., Bengs, C., Malmgren-Olsson, E. B., & Öhman, A. (2010). Young women facing multiple and intersecting stressors of modernity, gender orders and youth. *Social science & medicine*, *71*(9), 1567-1575. |
| **Hypothesis 3:** Cultural transmission of market-driven criterion is facilitated by evolutionary tendencies in young people to learn from older, successful and prestigious individuals (*prestige bias*) and to copy their peers. |
| Sources: evolutionary models and data  Barkow, J. H. (2014). Prestige and the ongoing process of culture revision. In *The psychology of social status* (pp. 29-45). Springer, New York, NY.  Barkow, J. H., O'Gorman, R., & Rendell, L. (2012). Are the new mass media subverting cultural transmission? *Review of General Psychology*, *16*(2), 121-133.  Henrich, J., & Gil-White, F. J. (2001). The evolution of prestige: Freely conferred deference as a mechanism for enhancing the benefits of cultural transmission. *Evolution and human behavior*, *22*(3), 165-196.  Jiménez, Á. V., & Mesoudi, A. (2019). Prestige-biased social learning: current evidence and outstanding questions. *Palgrave Communications*, *5*(1), 1-12.  Mesoudi, A. (2019). Cultural Evolution and Cultural Psychology. *Handbook of Cultural Psychology*. London:Guilford Press.  Mesoudi, A., Magid, K., & Hussain, D. (2016). How do people become WEIRD? Migration reveals the cultural transmission mechanisms underlying variation in psychological processes. *PloS one*, *11*(1).  Sources: Prestige bias, learning, celebrities and advertising and social media  Jiménez-Castillo, D., & Sánchez-Fernández, R. (2019). The role of digital influencers in brand recommendation: Examining their impact on engagement, expected value and purchase intention. *International Journal of Information Management*, *49*, 366-376.  Knoll, J., & Matthes, J. (2017). The effectiveness of celebrity endorsements: a meta-analysis. *Journal of the Academy of Marketing Science*, *45*(1), 55-75    Sources: Competitive peer networks and market-driven criterion  Abed, R., Mehta, S., Figueredo, A. J., Aldridge, S., Balson, H., Meyer, C., & Palmer, R. (2012). Eating disorders and intrasexual competition: Testing an evolutionary hypothesis amon young women. *The Scientific World Journal*, *2012*.  Banerjee, R., & Dittmar, H. (2008). Individual differences in children's materialism: The role of peer relations. *Personality and Social Psychology Bulletin*, *34*(1), 17-31.  Crone, E. A., & Dahl, R. E. (2012). Understanding adolescence as a period of social–affective engagement and goal flexibility. *Nature Reviews Neuroscience*, *13*(9), 636-650.  Demerath, P. (2009). *Producing success: The culture of personal advancement in an American high school*. Chicago, IL:University of Chicago Press.  de Vries, D. A., & Kühne, R. (2015). Facebook and self-perception: Individual susceptibility tonegative social comparison  on Facebook. *Personality and Individual Differences*, *86*, 217-221.  Fardouly, J., Pinkus, R. T., & Vartanian, L. R. (2017). The impact of appearance comparisons made through social media, traditional media, and in person in women’s everyday lives. *Body image*, *20*, 31-39.  Ferguson, C. J., Winegard, B., & Winegard, B. M. (2011). Who is the fairest one of all? How  evolution guides peer and media influences on female body dissatisfaction. *Review of General Psychology*, *15*(1), 11-28.  Gulas, C. S., & McKeage, K. (2000). Extending social comparison: An examination of the unintended consequences of  idealized advertising imagery. *Journal of Advertising*, *29*(2), 17-28.  Kim, H., Callan, M. J., Gheorghiu, A. I., & Matthews, W. J. (2017). Social comparison, personal relative deprivation, and materialism. *British Journal of Social Psychology*, *56*(2), 373-392.  Myers, T. A., & Crowther, J. H. (2009). Social comparison as a predictor of body dissatisfaction: A meta-analytic review.  *Journal of Abnormal Psychology*, *118*(4),683-698.  Patrick, H., Neighbors, C., & Knee, C. R. (2004). Appearance-related social comparisons: The role of contingent self-esteem and self-perceptions of attractiveness. *Personality and social psychology bulletin*, *30*(4), 501-514.  Rhee, J., & Johnson, K. K. (2012). Predicting adolescents' apparel brand preferences. *Journal of Product and Brand Management*, *21*(4), 255-264.  Roper, S., & Shah, B. (2007). Vulnerable consumers: the social impact of branding on children. *Equal Opportunities International*.  Spencer, R., Walsh, J., Liang, B., Mousseau, A. M. D., & Lund, T. J. (2018). Having it all? A qualitative examination of affluent adolescent girls’ perceptions of stress and their quests for success. *Journal of Adolescent Research*, 33(1), 3-33.  Luthar, S. S., & Kumar, N. L. (2018). Youth in high-achieving schools: Challenges to mental health and directions for evidence-based interventions. In *Handbook of School-Based Mental Health Promotion* (pp. 441-458). Springer, Cham.  Richins, M. L. (1995). Social comparison, advertising, and consumer discontent. *American Behavioral Scientist*, *38*(4),  593-607.  Vartanian, L. R., Hayward, L. E., Smyth, J. M., Paxton, S. J., & Touyz, S. W. (2018). Risk and resiliency factors related to body dissatisfaction and disordered eating: The identity disruption model. *International Journal of Eating Disorders*, *51*(4), 322-330.  Vogel, E. A., Rose, J. P., Okdie, B. M., Eckles, K., & Franz, B. (2015). Who compares and despairs? The effect of social comparison orientation on social media use and its outcomes. *Personality and Individual Differences*, *86*, 249-256. |
| **Hypothesis 3a: Young people’s self-displays of consumer goods are status and identity-enhancing signaling** |
| Sources: Consumer behaviour and evolutionary psychology:  Durante, K. M., & Griskevicius, V. (2016). Evolution and consumer behavior. *Current Opinion in Psychology*,*10*, 27-32.  Griskevicius, V. and Kendrick, D.T.(2013). 'Fundamental Motives: How Evolutionary Psychology Needs Influence Consumer Behavior.' Journal of Consumer Psychology, 23(3), pp. 372–386.  Miller, G. (2009). *Spent*. New York, NY: Viking Penguin.  Wang, Y., & Griskevicius, V. (2013). Conspicuous consumption, relationships, and rivals: Women's luxury products as signals to other women. *Journal of Consumer Research*, *40*(5), 834-854.  Sources: Consumer behaviour and your people’s social ecologies:  Dittmar, H., & Bond, R. (2010). I want it and I want it now: Using a temporal discounting paradigm to examine predictors of consumer impulsivity. *British Journal of Psychology*, *101*(4), 751-776  Elliott, R., & Leonard, C. (2004). Peer pressure and poverty: Exploring fashion brands and consumption symbolism among  children of the ‘British poor’. *Journal of Consumer Behaviour*, *3*(4), 347-359.  Furchheim, P., Jahn, S., & Zanger, C. (2013). When Altruism Is Perceived to Be Rare Would Materialists Buy Green?. *ACR North American Advances*.  Gil, L. A., Kwon, K. N., Good, L. K., & Johnson, L. W. (2012). Impact of self on attitudes toward luxury brands among  teens. *Journal of Business Research*, *65*(10), 1425-1433.  Roper, S., & Shah, B. (2007). Vulnerable consumers: the social impact of branding on children. *Equal Opportunities*  *International*, *26*(7), 712-728.  Shrum, L. J., Nancy Wong, Farrah Arif, Sunaina K. Chugani, Alexander Gunz, Tina M. Lowrey, Agnes Nairn et al. "Reconceptualizing materialism as identity goal pursuits: Functions, processes, and consequences." *Journal of Business Research* 66, no. 8 (2013): 1179-1185.  Shrum, L. J., Lowrey, T. M., Pandelaere, M., Ruvio, A. A., Gentina, E., Furchheim, P., ... & Nairn, A. (2014). Materialism: the good, the bad, and the ugly. *Journal of Marketing Management*, *30*(17-18), 1858-1881. |
| **Hypothesis 3b: Young people’s activity on social media often involves status and identity-enhancing signaling of market-driven criteria** |
| Sources: Evolutionary-informed theory about young people’s activity on social media  Donath, J. S. (2002). Identity and deception in the virtual community. In *Communities in cyberspace* (pp. 37-68). Routledge.  Donath, J. (2007). Signals, Truth, and Design. Cambridge, MA: MIT Press.  Sources: Signaling status and self-enhancement on social media  Liu, D., Ainsworth, S. E., & Baumeister, R. F. (2016). A meta-analysis of social networking  online and social capital. *Review of General Psychology*, *20* (4), 369-391.    Nesi, J., & Prinstein, M. J. (2019). In search of likes: Longitudinal associations between  adolescents’ digital status seeking and health-risk behaviors. *Journal of Clinical Child & Adolescent Psychology*, *48*(5), 740-748.  Pangrazio, L. (2019). Technologically situated: the tacit rules of platform participation. *Journal of Youth Studies*, 1-19.  Subrahmanyam, K., Reich, S. M., Waechter, N., & Espinoza, G. (2008). Online and offline social networks: Use of social networking sites by emerging adults. *Journal of applied developmental psychology*, *29*(6), 420-433.  Sources: Signaling self-enhancement through impression management on social media  Dorethy, M.D., Fiebert, M.S., & Warren, C.R. (2014). Examining social networking site  behaviors: Photo sharing and impression management on Facebook*. International Review of*  *Social Sciences and Humanities*, *6*(2), 111-116.  Liu, D., Ainsworth, S. E., & Baumeister, R. F. (2016). A meta-analysis of social networking  online and social capital. *Review of General Psychology*, *20* (4), 369-391.    Manago, A. M., Graham, M. B., Greenfield, P. M., & Salimkhan, G. (2008). Self-presentation  and gender on MySpace. *Journal of Applied Developmental Psychology*, *29*(6), 446-458.  Nesi, J., & Prinstein, M. J. (2019). In search of likes: Longitudinal associations between  adolescents’ digital status seeking and health-risk behaviors. *Journal of Clinical Child & Adolescent Psychology*, *48*(5), 740-748.  Pangrazio, L. (2019). Technologically situated: the tacit rules of platform participation. *Journal of Youth Studies*, 1-19.  Sibak, A. (2009). Constructing the self through the photo selection-Visual impression management on social networking websites. *Journal of Psychological Research on Cyberspace*, *3*(1) 1-6.  Subrahmanyam, K., Reich, S. M., Waechter, N., & Espinoza, G. (2008). Online and offline social networks: Use of social networking sites by emerging adults. *Journal of applied developmental psychology*, *29*(6), 420-433.  Young, K. (2009). Online social networking: An australian perspective. International Journal of Emerging Technologies and Society, 7(1), 39-57.  Zhao, S., Grasmuck, S., & Martin, J. (2008). Identity construction on Facebook: Digital empowerment in anchored relationships. *Computers in Human Behavior*, *24*(5), 1816-1836. |
